# Peptide binding to a bacterial signal peptidase visualized by peptide tethering and carrier-driven crystallization

**DOI:** 10.1107/S2052252515019971

**Published:** 2016-01-01

**Authors:** Yi Tian Ting, Paul W. R. Harris, Gaelle Batot, Margaret A. Brimble, Edward N. Baker, Paul G. Young

**Affiliations:** a School of Biological Sciences, The University of Auckland, Auckland 1142, New Zealand; b Maurice Wilkins Centre for Molecular Biodiscovery, The University of Auckland, Auckland 1142, New Zealand; c School of Chemical Sciences, The University of Auckland, Auckland 1142, New Zealand

**Keywords:** signal peptidase, Gram-positive pathogen, crystal structures, peptide complexes, peptide tethering, protein structure, enzyme mechanisms, structural biology

## Abstract

Bacterial type I signal peptidases (SPases) are membrane-anchored serine proteases that process the signal peptides of proteins exported *via* the Sec and Tat secretion systems. Despite their crucial importance for bacterial virulence and their attractiveness as drug targets, only one such enzyme, LepB from *Escherichia coli*, has been structurally characterized, and the transient nature of peptide binding has stymied attempts to directly visualize SPase–substrate complexes. Here, the crystal structure of SpsB, the type I signal peptidase from the Gram-positive pathogen *Staphylococcus aureus*, is reported, and a peptide-tethering strategy that exploits the use of carrier-driven crystallization is described. This enabled the determination of the crystal structures of three SpsB–peptide complexes, both with cleavable substrates and with an inhibitory peptide. SpsB–peptide interactions in these complexes are almost exclusively limited to the canonical signal-peptide motif Ala-*X*-Ala, for which clear specificity pockets are found. Minimal contacts are made outside this core, with the variable side chains of the peptides accommodated in shallow grooves or exposed faces. These results illustrate how high fidelity is retained despite broad sequence diversity, in a process that is vital for cell survival.

## Abstract 2

Utilizing a peptide-anchoring strategy, transient signal-peptide complexes of a Gram-positive bacterial signal peptidase were trapped, revealing the atomic details of their interaction.

## 1. Introduction   

Signal peptidases are membrane-bound proteolytic enzymes that play a crucial role in bacterial viability through their role in processing proteins that are translocated across biological membranes. This is achieved by their recognition and cleavage of a signal peptide located near the N-terminus of the substrate protein. Following cleavage of its signal peptide, the mature protein may then be secreted into the external medium or remain displayed on the bacterial surface. Such proteins are essential for the survival of bacteria in their particular bio­logical niches, and in the case of pathogenic bacteria are critically involved in infection and disease.

The type I signal peptidases (SPases) process the majority of proteins secreted by bacteria. They belong to a group of serine proteases that utilize a Ser–Lys catalytic dyad, which differs from the classical Ser–His–Asp triad found in most serine proteases and in the eukaryotic endoplasmic reticulum signal peptidase complex (SPC; Paetzel, Karla *et al.*, 2002 ▸). This difference from the eukaryotic SPC, and the accessibility of SPases at the bacterial cell surface, makes them an attractive target for the development of novel antibacterial agents. As membrane-bound proteins, however, the SPases have proved to be difficult subjects for structural analysis. Only one such enzyme, LepB from the Gram-negative *Escherichia coli*, has been structurally characterized to date (Paetzel *et al.*, 1998 ▸). Moreover, although LepB has been crystallized in complex with inhibitors, enabling peptide binding to be modelled (Paetzel *et al.*, 1998 ▸, 2004 ▸; Paetzel, 2014 ▸), the transient nature of SPase–peptide complexes has so far precluded the direct visualization of an SPase–substrate complex.

Signal peptides are extremely variable in sequence, but comprise three distinct regions: a positively charged N-terminal (N) region, a membrane-spanning hydrophobic (H) region and a polar C-terminal (C) region that precedes the mature protein sequence. The C region contains a strictly conserved Ala-*X*-Ala motif that is critical for cleavage (von Heijne, 1983 ▸); cleavage occurs immediately after the second Ala. Conservation of this motif led to the proposal of a −1,−3 rule, with the side chains of the two Ala residues predicted to occupy shallow pockets in the enzyme (von Heijne, 1983 ▸; Paetzel *et al.*, 1998 ▸). The −1 and −3 residues are also known as the P1 and P3 positions on the signal peptide, respectively, in accordance with the Schechter and Berger nomenclature (Schechter & Berger, 1967 ▸). Conversely, the residues of the mature, cleaved protein are numbered from P1′ immediately C-terminal to the cleavage site.

Mutational studies show an absolute requirement for small aliphatic side chains at positions P1 and P3 in the signal peptides of proteins exported *via* both the Sec and the Tat secretion systems (Fikes *et al.*, 1990 ▸; Shen *et al.*, 1991 ▸; Lüke *et al.*, 2009 ▸). Beyond the core Ala-*X*-Ala motif, however, almost any residue can apparently be tolerated (Shen *et al.*, 1991 ▸), except for proline at the P1′ position, which prevents signal-peptide cleavage (Barkocy-Gallagher & Bassford, 1992 ▸; Nilsson & von Heijne, 1992 ▸). Modelling into LepB shows that specificity pockets exist for the Ala residues at P1 and P3, but that most other signal-peptide side chains should be solvent-exposed, while the signal-peptide main chain could interact with β-strands that line the binding cleft (Paetzel, Dalbey *et al.*, 2002 ▸).

The human pathogen *Staphylococcus aureus* has only one active type I signal peptidase, SpsB. Like most other Gram-positive type I signal peptidases, SpsB has a relatively low sequence identity to the Gram-negative LepB (∼23%), and although it shares the key residues associated with catalysis and peptide binding in LepB (Paetzel, Karla *et al.*, 2002 ▸), its response to inhibitors is distinctly different. We therefore aimed to solve the structure of SpsB as the first example of a Gram-positive signal peptidase. To enable crystallization, we adopted a carrier-driven approach, crystallizing the extracellular domain of an SpsB catalytic mutant (S36A) as a maltose-binding protein (MBP) fusion construct, with the MBP molecule N-terminal to SpsB (Ting *et al.*, 2015 ▸). After unsuccessful attempts to crystallize SpsB–peptide complexes, we have now designed a tethering strategy that exploits the presence of the MBP partner and has enabled the capture of SPase–signal peptide complexes. Here, we report the details of this strategy and successful structural analyses of SpsB in complex both with cleavable signal-peptide substrates and with an inhibitor peptide, thus enabling direct visualization of the determinants of signal-peptide binding to type I SPases.

## 2. Experimental procedures   

### 2.1. Cloning and mutagenesis of SpsB   

The extracellular portions of SpsB and its S36A mutant were cloned and expressed as described previously (Ting *et al.*, 2015 ▸). Briefly, the *spsB* gene comprising the entire extracellular region of the protein, residues 26–191 (SpsB_26–191_), was PCR-amplified from *S. aureus* Newman strain (AP009351) genomic DNA and cloned into the vector pProExHta (Invitrogen). An S36A mutation was introduced by inverse PCR site-directed mutagenesis (Ochman *et al.*, 1988 ▸) to give an *spsB*-S36A construct. This was then subcloned into a modified MBP-pProExHta vector encoding an N-terminal maltose-binding protein (MBP) with an rTEV protease cleavage site prior to the multiple cloning cassette. Further modification by inverse PCR replaced the rTEV site with a three-residue linker (Ala-Gly-Ala) to give the final MBP-SpsB S36A construct reported previously (Ting *et al.*, 2015 ▸).

To generate a construct for the tethering of peptides, further modifications were made to both the *spsB* and *mbp* genes, again by inverse PCR site-directed mutagenesis. A high-fidelity DNA polymerase (*PfuUltra* II Fusion HS, Stratagene) was used for the PCR amplification of the MBP-SpsB S36A construct to produce a linearized PCR product with the desired mutation at the 5′-end of the sense primer. The template vector was removed by DpnI digestion, which digests only methylated DNA, and then re-circularized by intra­molecular ligation to produce a modified construct. In the first round of mutagenesis an MBP Q78C mutation was made to provide a free thiol group for covalent attachment of peptides. In the second round, an MBP K143G mutation was made to remove a potential clash between Lys143 of MBP and the peptide that was predicted from the uncomplexed SpsB crystal structure. Although the subsequent peptide-bound crystals adopted a different crystal-packing arrangement from that of the uncomplexed structure, making this mutation obsolete, the mutation was retained in the final construct. To facilitate the separation of full-length MBP-SpsB S36A from a minor MBP contaminant, the C-terminal residues 176–191 of SpsB, which were disordered in the uncomplexed structure, were replaced with a *Strep*-tag II (WSHPQFEK). Finally, the SpsB S36A moiety was reverted to active enzyme with the re-introduction by inverse PCR of the native Ser36 to produce the final MBP-SpsB^WT^-Stag construct. All mutants were sequence-verified.

### 2.2. Expression and purification   

Recombinant proteins were expressed and purified as described previously (Ting *et al.*, 2015 ▸). Briefly, recombinant protein expression was induced with IPTG (0.3 m*M*) at 37°C and the cells were lysed using a cell disruptor (Constant Cell Disruption Systems) at 124 MPa. After centrifugation to remove insoluble matter, the recombinant MBP-SpsB proteins were purified by immobilized metal-affinity chromatography (IMAC), concentrated and subjected to size-exclusion chromatography (SEC) on a Superdex S200 10/300 column (GE Healthcare) in crystallization buffer (10 m*M* Tris–HCl pH 8.0, 20 m*M* NaCl, 5 m*M* maltose). The proteins eluted in a single peak and were approximately 95% pure as indicated by SDS–PAGE, with a minor MBP contaminant. For the construct with the *Strep*-tag II, an additional purification step was used to remove contaminating MBP protein prior to SEC. Recombinant protein eluted from IMAC was then loaded onto a *Strep*-Tactin (IBA) column primed with 50 m*M* Tris–HCl pH 8.0, 500 m*M* NaCl, 5% glycerol, 500 m*M* imidazole. Nonspecifically binding protein was washed off the column with wash buffer (50 m*M* Tris–HCl pH 8.0, 150 m*M* NaCl, 1 m*M* EDTA). Finally, the protein with the *Strep*-tag II was eluted in wash buffer supplemented with 5 m*M* d-desthiobiotin.

### 2.3. Crystallization   

Crystallization conditions were identified by sitting-drop vapour diffusion as described previously (Ting *et al.*, 2015 ▸). MBP-SpsB S36A crystals were then optimized by hanging-drop vapour diffusion with multiple rounds of microseeding. The crystals used for X-ray data collection were grown at 18°C by mixing 1 µl protein solution (5 mg ml^−1^ in 10 m*M* Tris–HCl pH 8.0, 20 m*M* NaCl, 5 m*M* maltose) with 1 µl precipitant (12% PEG 8000, 20% ethylene glycol, 100 m*M* Tris–HCl pH 8.5).

The protein used for preparation of the peptide complexes was from the MBP-SpsB^WT^-Stag construct, in which the SpsB moiety was fully active, with residue 36 returned to the native Ser. Peptides for complex formation were synthesized by solid-phase peptide synthesis with an N-terminal *N*-bromo­acetyl moiety (Robey & Fields, 1989 ▸) for attachment to the engineered Cys residue on the MBP, and a C-terminal carboxyamide group. In each case, the peptide was solubilized in water and incubated with protein at a 25-fold or 50-fold molar excess at 4°C for 16 h. Under these conditions, the *N*-bromo­acetyl group reacted with Cys78 S^γ^ to give a thioether linkage. Co-crystals were successfully grown either directly from this peptide–protein mixture or after an additional SEC purification to remove unbound peptide. The extra SEC step made no apparent difference to crystal growth or the electron density, however, and the structures reported here were solved from crystals grown directly from the protein–peptide mixture. These crystals were grown by batch crystallization from a 1:1 mixture of the protein–peptide complex (10 mg ml^−1^ in 10 m*M* Tris–HCl pH 8.0, 20 m*M* NaCl, 5 m*M* maltose) with precipitant (12% PEG 8000, 20% ethylene glycol, 100 m*M* sodium acetate pH 5.3–5.5) in sitting drops under paraffin oil.

### 2.4. Data collection and structure determination   

Crystals were cryoprotected by adding an equal volume of cryoprotectant [precipitant containing 40%(*v*/*v*) ethylene glycol] directly onto the crystals prior to extraction from their mother liquor and were then flash-cooled in liquid nitrogen. X-ray diffraction data were recorded on the MX1 and MX2 beamlines of the Australian Synchrotron (AS) at a wavelength of 0.9537 Å at −162°C (McPhillips *et al.*, 2002 ▸). All data sets were integrated using *XDS* (Kabsch, 2010 ▸), re-indexed using *POINTLESS* (Evans, 2006 ▸) and scaled using *SCALA* (Evans, 2011 ▸). The MBP-SpsB S36A crystals belonged to the monoclinic space group *P*2_1_, with unit-cell parameters *a* = 57.7, *b* = 63.6, *c* = 79.9 Å, α = γ = 90, β = 92.6° and one MBP-SpsB S36A molecule per asymmetric unit. The MBP-SpsB^WT^-Stag peptide-conjugated crystals belonged to the orthorhombic space group *P*2_1_2_1_2_1_, with approximate unit-cell parameters *a* = 64.0, *b* = 80.2, *c* = 119.6 Å, α = β = γ = 90° (Table 1 ▸). The solvent volume of the crystals was calculated to be ∼52%, with a single MBP-SpsB–peptide complex in the asymmetric unit. The resolution cutoff for the structures was based on examining the values of *I*/σ(*I*) and *R*_p.i.m._ (Weiss, 2001 ▸) and the data correlation coefficient (CC_1/2_) values as described by Karplus & Diederichs (2012 ▸). The structure of the MBP-SpsB S36A fusion protein was determined by molecular replacement with *Phaser* (McCoy *et al.*, 2007 ▸) using the MBP structure (PDB entry 1anf; Quiocho *et al.*, 1997 ▸) as the search model, followed by autobuilding of the SpsB using *ARP*/*wARP* (Perrakis *et al.*, 1999 ▸). Both the thioether linkage between MBP Cys78 S^γ^ and the peptide, and the Ala-Gly-Ala linker between MBP and SpsB, were represented by excellent electron density. The structure was refined at 2.05 Å resolution using iterative cycles of manual building in *Coot* (Emsley *et al.*, 2010 ▸) and refinement with *REFMAC* (Murshudov *et al.*, 2011 ▸) to final values of *R* = 19.0% and *R*_free_ = 24.2%. The peptide-complex crystal structures were determined by molecular replacement using the refined MBP-SpsB S36A structure as a search model, and were refined as for the uncomplexed structure at resolutions of 1.9–2.1 Å. For these structures, the peptide was built manually into the model after refinement of the MBP-SpsB fusion-protein moiety was complete. Data-collection and refinement statistics are given in Table 1 ▸.

Model quality was monitored using *MolProbity* (Chen *et al.*, 2010 ▸). Ramachandran statistics for all structures show that >98% of the residues are in favoured positions, with no outliers. All figures were generated using *PyMOL* (v.1.5.0.4; Schrödinger). The coordinates and structure factors of the MBP-SpsB structures have been deposited in the Protein Data Bank under accession codes 4wvg, 4wvh, 4wvi and 4wvj.

## 3. Results   

### 3.1. Structure of SpsB   

*S. aureus* SpsB was expressed, purified and crystallized as an MBP fusion protein in which the N-terminal MBP was joined by a three-residue linker to the extracellular domain of SpsB (residues 26–191). In the uncomplexed structure the catalytic Ser36 of SpsB was mutated to Ala (Ting *et al.*, 2015 ▸), but this mutation has no effect on the structure of the SpsB moiety, which is essentially identical (r.m.s.d. of <0.2 Å over 153 aligned C^α^ atoms) in the SpsB–peptide structures described later, in which residue 36 is restored to the catalytically active Ser.

**Figure 1:**
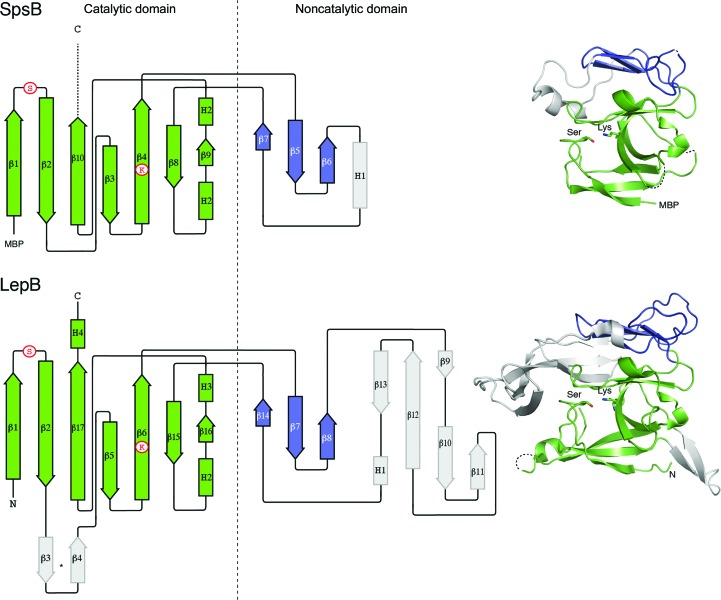
Structures of SpsB and LepB. The topology diagrams on the left are colour-coded as for the adjacent ribbon diagrams. The conserved catalytic domains are in green, whereas the noncatalytic domain has a core three-stranded β-sheet (blue) flanked by a highly divergent region (grey). Dashed lines represent regions that are not visible in the electron density. The positions of key catalytic residues are shown in circles or in stick form in the ribbon diagrams. N, N-terminus; C, C-terminus; MBP, maltose-binding protein. The LepB structure is from PDB entry 1b12 (Paetzel *et al.*, 1998 ▸).

The crystal structures determined here revealed a two-domain SpsB fold that is homologous to that of *E. coli* LepB (Paetzel *et al.*, 1998 ▸), but like most Gram-positive SPases is substantially truncated compared with the *E. coli* enzyme (166 residues compared with 247; Fig. 1 ▸). There are some differences in the catalytic domain (SpsB residues 26–84 and 138–191), where SpsB lacks one prominent β-hairpin loop and a C-terminal helix that are present in LepB, but the major differences are in the second, noncatalytic domain (residues 85–137) in SpsB. This domain is much more divergent and has little sequence or structural homology, with only a small three-stranded β-sheet in common between SpsB and LepB. This domain has no known function, although it has been suggested to contribute to the recognition of the mature protein portion of signal peptides (Choo & Ranganathan, 2008 ▸).

**Figure 2:**
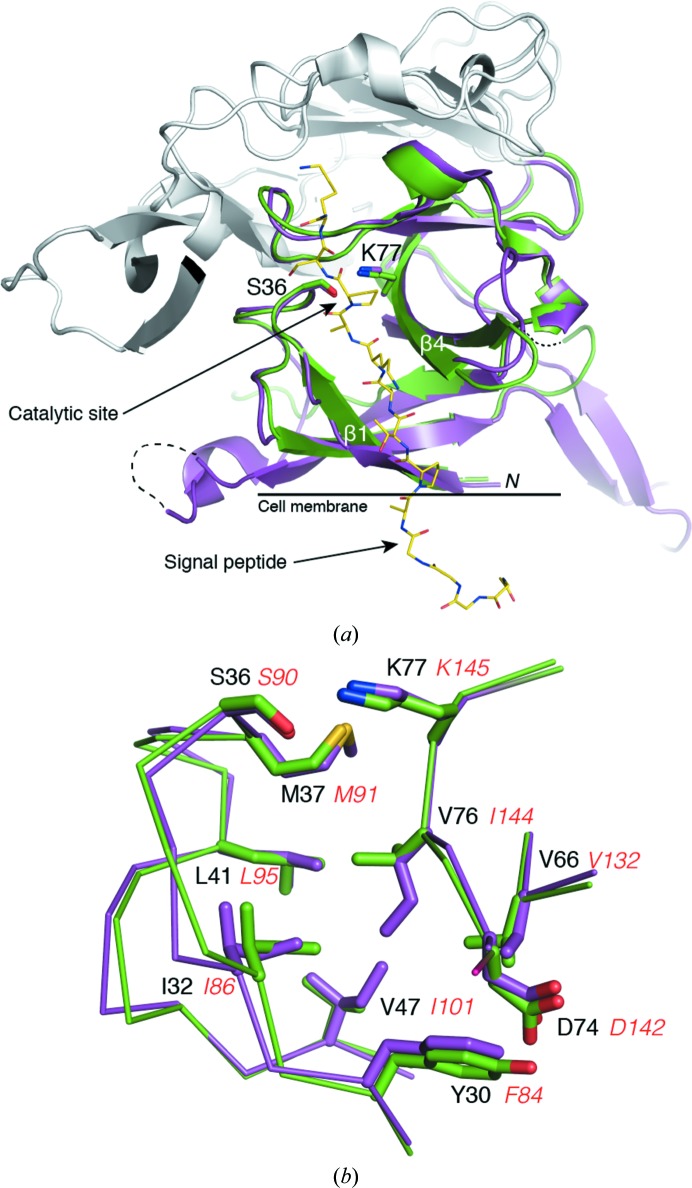
Structural comparison between SpsB and LepB. (*a*) Cartoon showing the conserved extracellular catalytic domains of SpsB (green; Pep3 complex) and LepB (magenta; PDB entry 1b12). The catalytic Ser and Lys residues (in stick form) are shown at the head of the peptide-binding cleft. The peptide Pep3 bound to SpsB is shown in yellow stick form. The divergent noncatalytic domains are shown in grey. Dashed lines represent parts of the protein that are not visible in the electron density. (*b*) Close-up view of the residues that form the S1 and S3 pockets of the peptide-binding cleft of SpsB and LepB. SpsB residues are labelled in black, while the corresponding LepB residues are shown in red italics.

Crucially, the core of the SpsB catalytic domain matches LepB very closely (Fig. 2 ▸ *a*), with an r.m.s.d. of 0.72 Å over 82 aligned C^α^ atoms. The catalytic residues Ser36 and Lys77 (Ser77 and Lys145 in LepB) are similarly arranged at the head of the peptide-binding cleft, and the hydrophobic residues that line the cleft are highly conserved and virtually identically arranged (Fig. 2 ▸ *b*).

### 3.2. Peptide complexes   

**Figure 3:**
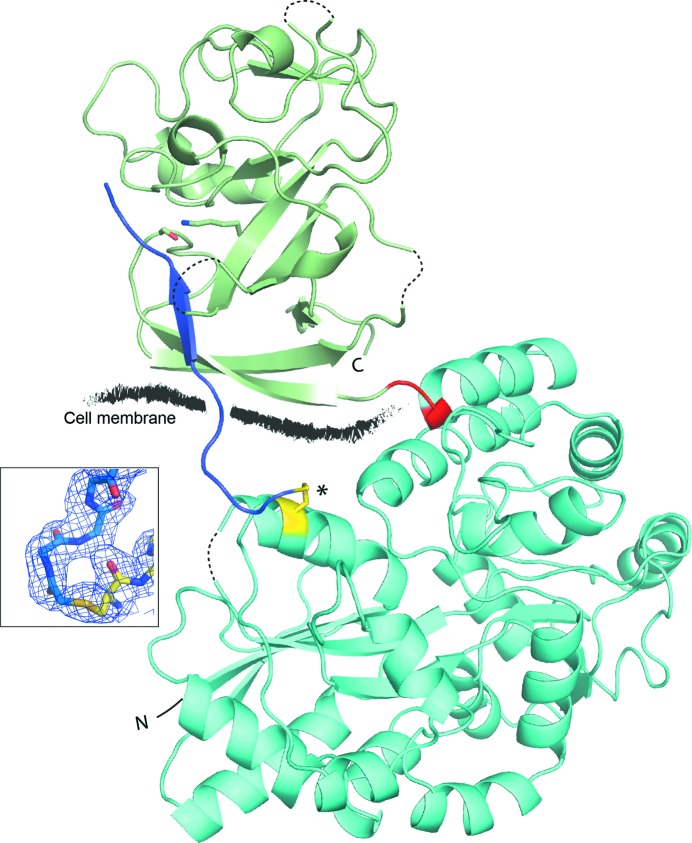
Structure of an MBP-SpsB–inhibitor peptide complex. Cartoon diagram showing the overall structure of a representative MBP-SpsB–inhibitor peptide complex. MBP is coloured cyan, SpsB green and the peptide blue. The three-amino-acid linker (Ala-Gly-Ala) between MBP and SpsB is shown in red and the engineered thiol group (MBP Q78C) in yellow (marked with an asterisk). Shown in stick form (green), adjacent to the peptide, are the SpsB catalytic residues Ser36 and Lys77. The N- and C-­termini of the fusion protein are designated N and C, respectively. Disordered loops are shown as dashed lines. The stylized black line shows where the cell membrane would be relative to SpsB and the signal peptide *in vivo*. The inset shows a view of the thioether bond linking the N-­terminus of the Pep3 peptide (blue) to the engineered cysteine residue (Cys78, yellow) on MBP. Residues are encompassed by 2*F*_o_ − *F*_c_ electron density contoured at 1.0σ, which is orientated to clearly show the continuous electron density between the peptide and Cys78. The peptide position is not constrained by crystal contacts. There is no interaction between the peptide and any adjacent monomers in the crystal lattice, with the nearest adjacent monomer ∼14 Å from the peptide.

As co-crystallization experiments with signal peptides consistently failed to show bound peptide, we modified the MBP fusion construct to allow covalent attachment of peptide substrates. The peptides used were selected based on their efficient cleavage by, or inhibition of, SpsB activity (Bruton *et al.*, 2003 ▸) and were synthesized with an *N*-bromoacetyl moiety that formed a covalent thioether linkage to a strategically positioned thiol group on MBP, which had been introduced by mutating Gln78 to Cys (Fig. 3 ▸). Peptides with variable numbers of residues between the *N*-bromoacetyl moiety and the signal-peptide C region were tested to optimize the peptide positioning in the context of a rigid crystal structure, with a four-amino-acid sequence (GGGG) at the peptide N-terminus resulting in the appropriate positioning of the peptide C region in the SpsB peptide-binding cleft. This construct mimics the binding of an intact signal peptide, the H region of which would be embedded in the cell membrane, and ensured that the peptide would be presented in the correct orientation to the peptide-binding cleft of SpsB. SpsB was also restored to its active form by mutating residue 36 back to the native Ser, and the C-terminal residues 176–191, which were disordered in the uncomplexed structure, were replaced with a *Strep*-tag II to aid purification.

Using this tethering strategy, SpsB was crystallized with three different peptides (Table 2 ▸). Two (Pep1 and Pep2) are substrate peptides with very different C-region sequences which are actively cleaved by our SpsB constructs (Ting *et al.*, 2015 ▸; Bruton *et al.*, 2003 ▸). In the Pep1 and Pep2 substrate complexes cleavage has occurred between P1 and P1′, releasing the mature protein sequence (residues P1′–P3′). The third peptide, Pep3, is similar to Pep2 but has proline at P1′, making it an inhibitor that remains uncleaved in the complex (Bruton *et al.*, 2003 ▸).

**Figure 4:**
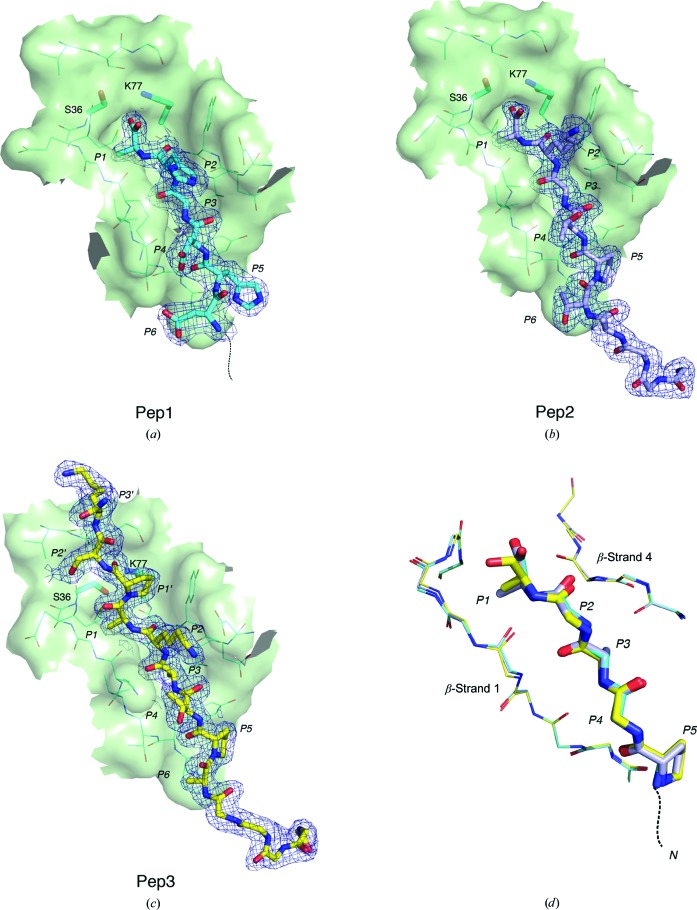
Conserved binding mode of the signal-peptide C regions. (*a*), (*b*) and (*c*) show the peptide-binding cleft in surface representation with the peptides Pep1, Pep2 and Pep3 shown in stick mode in their 2*F*_o_ − *F*_c_ electron density contoured at 1.0σ. Peptide C-region residues are labelled P1–P6. (*a*) The structure of Pep1 shows an absence of electron density for the mature peptide region (P1′–P3′), which has been cleaved by SpsB. The catalytic Ser is rotated away from the carboxylate group of the cleaved peptide. There is no interpretable electron density for the peptide between P7 and the MBP linkage. (*b*) As for Pep1, the Pep2 peptide structure shows no electron density for the mature peptide region and rotation of the catalytic Ser away from the carboxylate group of the cleaved peptide. The entire cleaved peptide from P1 to the MBP linkage is well ordered. (*c*) The Pep3 inhibitor (yellow) bound in the peptide-binding cleft. A proline at position P1′ prevents cleavage. (*d*) Overlay of Pep1 (cyan), Pep2 (blue) and Pep3 (yellow) signal peptides shows that all peptide and SpsB residues (β-strands 1 and 4) share virtually identical main-chain atom positions, with only the Pep3 P1 residue being significantly displaced. The peptides (P1–P5) are shown in stick form and the SpsB residues as lines. All side chains have been removed, except for the Ala residues at P1 and P3 and Pro at P5.

**Figure 5:**
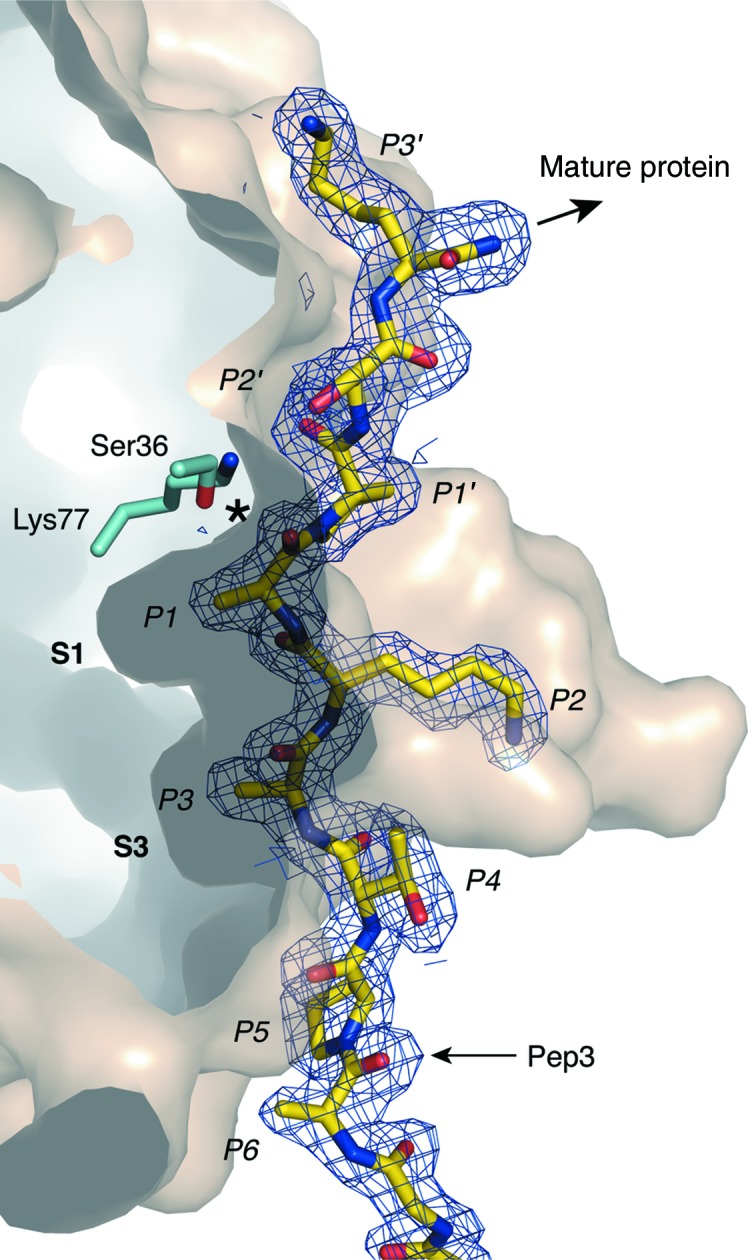
Specificity of SpsB for the signal-peptide C region. (*a*) Surface representation contoured to show the S1 and S3 pockets, which contain Ala side chains at P1 and P3 of the peptide. The peptide inhibitor Pep3 (yellow) is shown in its 2*F*_o_ − *F*_c_ electron density contoured at 1.0σ, with its C-region residues labelled P1–P6 and P1′–P3′ (mature protein portion). The catalytic serine and lysine residues are in cyan adjacent to the cleavage site (marked with an asterisk).

All three SpsB–peptide complex structures were determined at resolutions of 1.9–2.1 Å, with well defined electron density for the peptides over the entire binding region (Fig. 4 ▸). In each structure the peptide occupies a shallow cleft between SpsB β-strands 1 and 4, with the catalytic residues situated at the head of the cleft (Figs. 4 ▸ and 5 ▸). Residues P2–P5 of all three peptides bind in an identical fashion, and all SpsB residues that contact Pep1 and Pep2 are also identically positioned [r.m.s.d. of 0.09 Å over 72 main-chain atoms from the peptide (P1–P5) and SpsB β-strands 1 and 4; Fig. 4 ▸ *d*]. The Ala side chains of peptide residues P1 and P3 occupy hydrophobic depressions that constitute the S1 and S3 specificity pockets, respectively (Fig. 5 ▸). In the Pep1 and Pep2 substrate complexes, the P1 methyl group makes intimate contacts with the side chains of Ile32, Met37 and Val76 in the S1 pocket, with main-chain atoms from Lys33, Gly34, Ser36 and Met37 also at a van der Waals distance. In the Pep3 structure, the P1 methyl group does not protrude as far into the S1 pocket, with an average distance of 4.1 Å between the P1 methyl group and the residues of the S1 pocket, compared with 3.8 Å for Pep1 and Pep2. This is likely to be because the rigid proline pyrrolidine ring at the P1′ position prevents the P1 residue from further approaching the enzyme. The S3 pocket is flanked by Tyr30, Leu41, Val66 and Asp74 C^β^, while Ile32 and Val76 form a bridge between the two pockets, as noted for LepB (Paetzel *et al.*, 1998 ▸). The S3 pocket is broader, with an average distance between the P3 methyl group and the residues that line the S3 pocket for all three peptide structures of 4.2 Å. This is consistent with observed signal-peptide sequence variability at P3, which allows the pocket to accommodate larger aliphatic residues.

**Figure 6:**
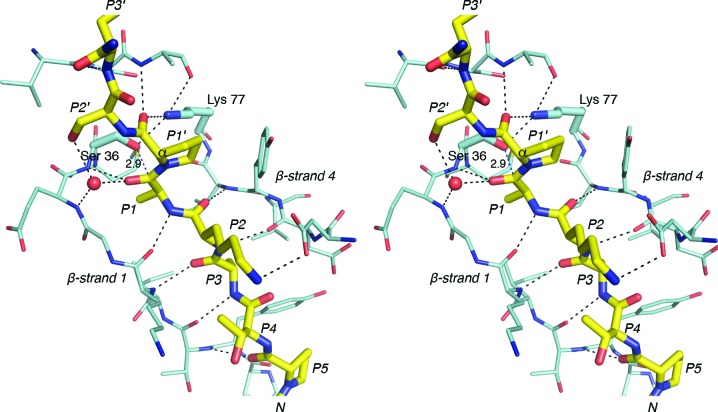
Hydrogen-bond contacts between the signal peptide and SpsB. A stereo figure showing an inhibitory signal peptide (yellow) bound to SpsB. The peptide makes main-chain parallel β-­sheet hydrogen-bond interactions (dashes) with strands that line the peptide-binding cleft, but makes no contact with SpsB before residue P5 or after residue P3′. The side chain of Ser36 is directed at the plane of the P1 scissile bond, with its O^γ^ atom 2.9 Å from the carbonyl C atom. The rigid P1′ proline pyrrolidine ring C^α^ atom prevents the P1 carbonyl C atom from moving closer to Ser36. Water is shown as a red sphere.

The most substantial peptide–enzyme interaction is thus mediated through the core Ala-*X*-Ala residues, which also make parallel β-sheet hydrogen bonds to β-strands 1 and 4 that flank the binding cleft (Fig. 6 ▸). β-Strand interactions between peptide and enzyme are common in substrate–protease complexes (Tyndall *et al.*, 2005 ▸). The side chains of the P2 and P4 peptide residues remain solvent-exposed and make no contact with the enzyme, in accordance with predictions from the LepB structure (Paetzel *et al.*, 1998 ▸; Paetzel, Dalbey *et al.*, 2002 ▸). Only one other hydrogen bond is apparent, linking the P5 proline carbonyl O atom to the peptide N atom of Thr31. The positioning of the proline orientates the peptide chain so that no further residues make main-chain contacts with the enzyme (Fig. 5 ▸). The Pep1 structure shows that the peptide is similarly directed away from the enzyme at P5 when the residue is histidine (Fig. 4 ▸).

In the peptide-substrate complexes, cleavage has occurred between P1 and P1′, releasing the mature protein sequence (residues P1′–P3′). The SpsB construct used here had previously been shown to be fully active, cleaving signal peptides to release a mature protein (Ting *et al.*, 2015 ▸) and cleaving peptides with C-terminal fluorescent tags to release the tag. This implies that the lack of density for the mature protein portion of the cleavable peptides Pep1 and Pep2 is owing to enzymatic cleavage and not to disorder of the peptide in the crystal structure. In this post-cleavage state the C-terminal carboxyl group of P1 is hydrogen-bonded to the SpsB general base (Lys77) and receives a probable C—H⋯O hydrogen bond from C^β^ of the nucleophilic Ser36 (C⋯O distance 2.8 Å, C^α^—C^β^⋯O angle 104°). The Ser36 hydroxyl points away from the cleaved peptide, which is the result of a clash with the C-terminus of the cleaved peptide (Fig. 4 ▸).

In contrast, in the SpsB–inhibitor crystal structure the peptide remains intact, with no cleavage between P1 and P1′. The hydroxyl group of the nucleophilic Ser36 is directed towards the plane of the P1–P1′ scissile bond, 2.9 Å from the carbonyl C atom, but the rigid P1′ proline pyrrolidine ring prevents further approach. The P1 carbonyl O atom makes two hydrogen bonds (Fig. 6 ▸): to the main-chain NH of Ser36, the main contributor to the expected ‘oxyanion hole’, and to a water molecule that bridges to the main-chain NH of Glu35. This water molecule occupies the position of Ser88 O^γ^ in LepB, which has been implicated in stabilizing the tetrahedral intermediate in this protein (Paetzel *et al.*, 1998 ▸; Carlos *et al.*, 2000 ▸). Protein–peptide interactions in the active site support the catalytic mechanism proposed for SPases (Paetzel *et al.*, 1998 ▸), which involves attack by the nucleophilic serine on the *si* face of the scissile peptide bond. Interactions between the mature protein portion of this peptide and SpsB are limited to P1′ and P3′. The P1′ proline carbonyl O atom is hydrogen-bonded to the ∊-amino group of Lys77 and the main-chain NH of Lys153, and the P3′ main-chain NH hydrogen-bonds to the carbonyl O atom of Val151, with the P3′ side chain positioned across the upper catalytic domain surface. Any additional residues in the mature portion of the pre-protein should project away from the enzyme.

## 4. Discussion   

Signal peptides are critical N-terminal extensions that function as an address code for proteins, with cleavage of the signal peptide by a signal peptidase the last step in protein secretion. Type I signal peptidases all recognize a central Ala-*X*-Ala motif that precedes the cleavage site, but the sequence diversity outside this canonical motif is such that ambiguity remains as to how the enzyme can process hundreds of different peptides while retaining strict fidelity. A complicating factor is that enzymes from different bacteria have varied susceptibility to SPase I inhibitors. Thus, although SpsB and LepB were predicted to share a conserved catalytic apparatus, LepB inhibitors show only limited inhibition of SpsB (Paetzel *et al.*, 1998 ▸; Smith *et al.*, 2010 ▸; Smith & Romesberg, 2012 ▸), suggesting structural differences between Gram-positive and Gram-negative bacterial signal peptidases.

The structure of SpsB, the first from a Gram-positive bacterium and the second SPase I to be crystallized, shows that the Gram-positive SpsB and the Gram-negative LepB have a conserved fold despite their limited sequence identity (23%). While there is a large variation in the size and structure of the noncatalytic domain, the core catalytic domains are very similar. Importantly, the peptide-binding clefts of the two enzymes are virtually identical and, as predicted by the amino-acid sequence, have matching catalytic apparatus. In the present study, the SpsB–inhibitor peptide (Pep3) complex represents a model for the pre-protein–enzyme Michaelis complex, whereas the substrate peptides (Pep1 and Pep2) give a picture of the post-cleavage state. There is little change in the peptide-binding cleft between the uncomplexed and peptide-bound SpsB structures. In the uncomplexed structure Tyr30 partially occupies the S3 pocket but moves when peptides bind, and Val76, which forms a bridge between the S1 and S3 pockets, adopts an alternative rotamer. These changes are consistent with changes observed between the apo and inhibitor-bound structures of *E. coli* LepB (Paetzel, Dalbey *et al.*, 2002 ▸), with the homologous residues in SpsB adopting the same positions as in LepB.

Protein–peptide interactions in SpsB support the proposed catalytic mechanism for SPases, with nucleophilic attack by the serine (Ser36) on the *si* face of the scissile peptide bond (Paetzel *et al.*, 1998 ▸). In Pep3, the proline at P1′ prevents cleavage, both for steric reasons and because proline, a secondary amine, cannot readily accept a proton in acid–base catalysis (Barkocy-Gallagher & Bassford, 1992 ▸). Like LepB, the SpsB peptide-binding cleft contains two shallow pockets, designated the S1 and S3 substrate-binding pockets, which accommodate the methyl side chains of the P1 and P3 Ala residues. The residues that line these pockets are remarkably conserved between SpsB and LepB, consistent with their common Ala-*X*-Ala specificity (Fig. 2 ▸ *b*). The high level of conservation in and around the two pockets is also consistent with peptide-binding studies showing that alteration of the residues that bridge between the two pockets in LepB leads to slippage in the site of peptide cleavage and alteration of the S3 pocket specificity (Karla *et al.*, 2005 ▸; Ekici *et al.*, 2006 ▸). In our structures the side chains of the P2 and P4 peptide residues remain solvent-exposed. This contradicts predictions from *in silico* modelling of LepB (Choo *et al.*, 2008 ▸), in which all three side chains of the Ala-*X*-Ala motif (P1, P2 and P3) are buried in the binding groove, and confirms the conventional view of peptide binding (Paetzel, Dalbey *et al.*, 2002 ▸).

Our SpsB–signal peptide structures show clearly that the peptides form parallel β-strand interactions with strands 1 and 4, which line the peptide-binding cleft of SpsB. Extended β-strand conformations such as this appear to be an almost universal feature of peptide binding to proteases (Tyndall *et al.*, 2005 ▸). Significantly, it is the core Ala-*X*-Ala motif that provides the most substantial peptide–enzyme interaction (Fig. 6 ▸), and the only residues outside this core to make main-chain hydrogen-bonding contacts are at P5, and at P1′ and P3′ in the mature portion of the peptide. In the Pep2 and Pep3 structures, the P5 proline directs the peptide chain away from the enzyme, with no residues prior to P5 making contact with the enzyme. A proline is commonly found at P5 in *S. aureus* signal peptides and at P5 and P6 in signal peptides from other bacteria (Bruton *et al.*, 2003 ▸; Choo & Ranganathan, 2008 ▸; Schallenberger *et al.*, 2012 ▸). This proline, at the boundary between the H and C regions of signal peptides, had been predicted to influence the conformational change from α-helix to β-strand (von Heijne, 1990 ▸). In Pep1, however, the P5 residue is histidine and this peptide is similarly bent away from SpsB, implying that signal peptides bind similarly regardless of sequence, with main-chain contacts between peptide and SpsB restricted to peptide residues P5–P3′. Conversely, the orientation of the P3′ side chain in the Pep3 structure suggests that no further residues in the mature portion of the pre-protein are likely to influence peptide binding; only the nine peptide residues between P6 and P3′ are in close proximity to the enzyme.

A number of factors in the enzyme–peptide substrate complex suggest that it is optimized to enhance product turnover. These include the minimal degree of interaction between the peptide and enzyme and the conserved binding mode of different peptide substrates. The shallow nature of the substrate-binding cleft, in which the main-chain atoms of the peptide substrate remain solvent-exposed, should also promote rapid association of substrates and dissociation of products, as required for multiple rapid turnovers (Tyndall *et al.*, 2005 ▸).

**Figure 7:**
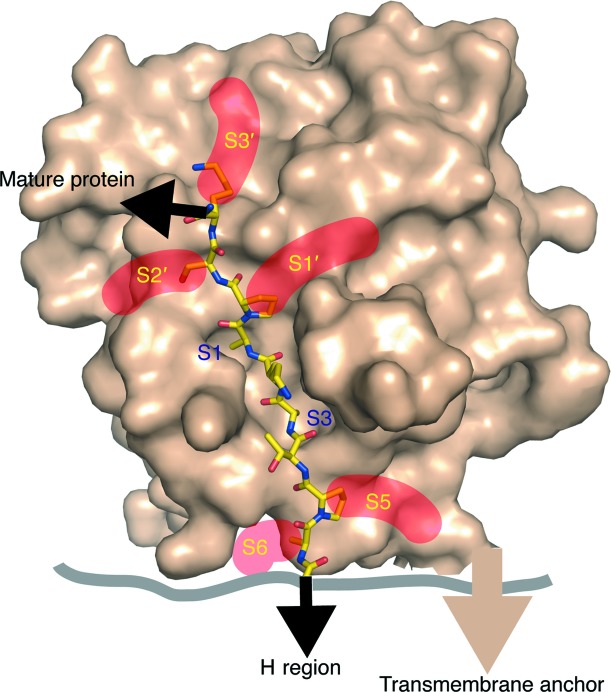
General model of signal-peptide binding to SpsB. A surface view showing that the enzyme accommodates the diverse side chains of signal peptides in shallow grooves or on exposed faces (subsites S5, S6 and S1′–S3′), with only the alanine side chains of the canonical Ala-*X*-Ala buried in the S1 and S3 pockets. The core Ala-*X*-Ala motif both defines specificity and accounts for the majority of interactions between the peptide and the enzyme, while the ‘subsites’ are such that wide peptide diversity can be accommodated.

Looking beyond the P1 and P3 residues, which dominate the peptide-binding interactions and dictate the position of the peptide in the peptide-binding cleft, the P2 and P4 side chains point directly out into the solvent, while the P5 and P6 side chains are accommodated by shallow grooves. Likewise, the diverse side chains of the P1′, P2′ and P3′ peptide residues are accommodated by depressions in the enzyme surface (Fig. 7 ▸). These grooves or exposed faces allow the enzyme to bind peptides with highly diverse side-chain composition without affecting fidelity, which is dependent on the Ala-*X*-Ala motif. The structures presented here, together with signal-peptide sequence and mutagenesis data (Jain *et al.*, 1994 ▸; Dev *et al.*, 1990 ▸; von Heijne, 1990 ▸; van Dijl *et al.*, 1995 ▸; Karla *et al.*, 2005 ▸; Ekici *et al.*, 2006 ▸; Shen *et al.*, 1991 ▸), thus suggest that the primary role of these ‘subsites’ is to accommodate the diversity of signal-peptide side chains.

While it is not clear whether the cleavage of signal peptides occurs during or after protein translocation, the anchoring of the H region of signal peptides in the membrane appears to be essential for orientating the signal peptide. Only when we were able to mimic this restraint by tethering the N-terminus of our peptide could we successfully co-crystallize SpsB with bound peptides. Gram-positive signal peptides are longer than both Gram-negative bacterial and eukaryotic signal peptides, with the site of cleavage predicted to be on the membrane surface (Dalbey *et al.*, 2012 ▸; Choo & Ranganathan, 2008 ▸). Whether cleavage occurs at the cell surface, as modelled here (Fig. 7 ▸), or within the confines of the membrane, as predicted for LepB, remains uncertain. However, from our structures we can infer a minimalistic model of peptide recognition in which the core Ala-*X*-Ala motif both defines specificity and accounts for the majority of the interactions between the peptide and enzyme. Few other residues make specific contacts, and these involve main-chain atoms, independent of sequence, with the divergent side chains accommodated *via* exposed faces. 

## Supplementary Material

PDB reference: apo SpsB, 4wvg

PDB reference: SpsB–Pep1, 4wvh

PDB reference: SpsB–Pep2, 4wvi

PDB reference: SpsB–Pep3, 4wvj

## Figures and Tables

**Table 1:** Data-collection and refinement statistics Values in parentheses are for the outermost shell.

	SpsB S36A apoenzyme	SpsB–Pep1	SpsB–Pep2	SpsB–Pep3
Data collection				
X-ray source	MX1, AS	MX1, AS	MX2, AS	MX2, AS
Wavelength	0.9537	0.9537	0.9537	0.9537
Resolution (Å)	19.44–2.05 (2.11–2.05)	19.77–2.10 (2.16–2.10)	19.78–1.90 (1.94–1.90)	19.75–1.95 (2.00–1.95)
Space group	*P*2_1_	*P*2_1_2_1_2_1_	*P*2_1_2_1_2_1_	*P*2_1_2_1_2_1_
Unit-cell parameters				
*a* (Å)	57.68	63.66	63.99	64.11
*b* (Å)	63.56	80.19	80.23	80.10
*c* (Å)	79.89	119.40	119.88	119.65
α = γ (°)	90.00	90.00	90.00	90.00
β (°)	92.59	90.00	90.00	90.00
Total reflections	275184 (21095)	534471 (43560)	466778 (31178)	551863 (39048)
Unique reflections	36318 (2779)	36431 (2945)	49376 (3283)	45665 (3190)
Multiplicity	7.6 (7.6)	14.7 (14.8)	9.5 (9.5)	12.1 (12.2)
Completeness (%)	99.8 (99.8)	99.9 (100.0)	99.9 (100.0)	99.9 (100.0)
*R*_p.i.m._ †	0.082 (0.608)	0.119 (0.983)	0.090 (0.964)	0.099 (0.818)
〈*I*/σ(*I*)〉	10.1 (1.4)	10.0 (1.6)	11.1 (1.5)	10.3 (1.5)
CC_1/2_ ‡	0.994 (0.528)	0.994 (0.589)	0.997 (0.514)	0.998 (0.482)
Refinement				
Resolution (Å)	79.81–2.05	66.58–2.10	66.68–1.90	66.56–1.95
*R*_work_/*R*_free_	0.190/0.242	0.205/0.248	0.188/0.220	0.185/0.218
No. of atoms				
Protein	3977	4030	4022	3991
Peptide ligand	0	47	58	80
Maltose	23	23	23	23
Water	356	248	371	374
Average *B* factors (Å^2^)				
Protein	29.35	29.15	24.27	26.55
Peptide ligand	0	39.32	31.23	29.73
Maltose	18.84	19.82	16.65	19.33
Water	35.09	33.51	33.17	36.82
R.m.s. deviations				
Bond lengths (Å)	0.007	0.007	0.008	0.008
Bond angles (°)	1.16	1.17	1.22	1.27
Ramachandran favoured (%)	98.2	98.5	98.1	98.66
Ramachandran outliers (%)	0	0	0	0
PDB code	4wvg	4wvh	4wvi	4wvj

† Precision-indicating *R* factor (see Weiss, 2001 ▸).

‡ Correlation coefficient (see Karplus & Diederichs, 2012 ▸).

**Table 2:** Peptide sequences

Peptide	Amino-acid sequence														
							P6	P5	P4	P3	P2	P1	P1′	P2′	P3′
Pep1	BrAc–	G	G	G	G	A	D	H	D	A	H	A	S	E	T
Pep2	BrAc–	G	G	G	G	A	V	P	T	A	K	A	A	S	K
Pep3	BrAc–	G	G	G	G	G	A	P	T	A	K	A	P	S	K
